# The inhibition of necroptosis prevents neuronal death in a mouse model of Alzheimer’s disease pathology

**DOI:** 10.1002/alz.087322

**Published:** 2025-01-03

**Authors:** Sebastiaan Moonen, Marta J Koper, Alicja Ronisz, Simona Ospitalieri, Zsuzsanna Vegh, Dries T’Syen, Sabine Rabe, Matthias Staufenbiel, Bart De Strooper, Sriram Balusu, Dietmar Rudolf Thal

**Affiliations:** ^1^ Laboratory for the Research of Neurodegenerative Diseases, KU Leuven‐VIB CBD, Leuven Belgium; ^2^ Laboratory for Neuropathology, KU Leuven, Leuven Belgium; ^3^ Laboratory for Biological Psychology, KU Leuven, Leuven Belgium; ^4^ Novartis Institutes for Biomedical Sciences, Basel Switzerland; ^5^ Hertie Institute for Clinical Brain Research, Tuebingen Germany; ^6^ UK Dementia Research Institute, University College London, London United Kingdom; ^7^ UZ Leuven, Leuven Belgium

## Abstract

**Background:**

As neurodegenerative diseases advance, postmitotic neurons are affected by disturbed proteostasis and the accumulation of misfolded proteins. This renders neurons sensitive to cell death, ultimately leading to progressive neuron loss. Multiple studies show the involvement of distinct pathways of regulated cell death (RCD) in neurodegenerative diseases, such as necroptosis. In human AD, activation of necroptosis has been shown to contribute to the neurodegenerative process. However, despite considerable research efforts, it is still not clear which mechanism exactly underlies neuronal loss in AD.

**Methods:**

We studied necroptosis activation in mouse models of AD pathology and primary mouse neurons after the addition of pTau seeds. Immunohistochemistry was used to study neuronal loss and the presence of the activated necroptosis‐related proteins pRIPK1, pRIPK3 and pMLKL. TAU22 and TAU58 mice were used to assess the influence of Tau pathology, APP23 for amyloid pathology, and APP23xTAU58 for both Tau and amyloid pathology, representing more closely human AD neuropathological changes. APP23xTAU58 mice were subjected to intraperitoneal injection of the necroptosis‐inhibiting drugs dabrafenib or ponatinib from two to six months old, followed by neuropathological assessment and behavior testing using the social preference and social novelty test, T‐maze, and fear conditioning.

**Result:**

Necroptosis activation was detected in mice developing Tau pathology and in primary mouse neurons stimulated with AD pTau seeds. APP23xTAU58 mice showed the highest necroptosis activation, while no significant upregulation was observed in APP23 mice. The necroptosis‐related proteins (pRIPK1, pRIPK3, pMLKL) were present in neuronal granulovacuolar degeneration (GVD) bodies, predominantly in CA1 and subiculum of the hippocampus, entorhinal cortex, amygdala, parietal and frontal cortex. In the subiculum and amygdala, the number of neurons showing pMLKL‐positive GVD lesions was associated with neuronal loss, as seen in human AD. Both dabrafenib and ponatinib reduced the number of neurons showing pMLKL‐positive GVD bodies, prevented neuronal loss, and ameliorated social recognition memory.

**Conclusion:**

Taken together, these results point to “GVD‐necroptosis”, as a presumably delayed form of necroptosis, and a mechanism underlying AD pathology‐related neuronal loss. Necroptosis inhibition as shown here in APP23xTAU58 mice is a novel approach to ultimately prevent “GVD‐necroptosis” induced neuronal death.

**Funding**: SAO/FRA and FWO.

